# Schwannoma with an Uncommon Upper Lip Location and Literature Review

**DOI:** 10.1155/2013/363049

**Published:** 2013-02-26

**Authors:** Tuba Bayindir, M. Tayyar Kalcioglu, Mehmet T. Cicek, Nese Karadag, Abdurrahman Karaman

**Affiliations:** ^1^Department of Otorhinolaryngology, Medical Faculty, Inonu University, 34347 Malatya, Turkey; ^2^Department of Otorhinolaryngology, Medical Faculty, Istanbul Medeniyet University, 34347 Istanbul, Turkey; ^3^Department of Pathology, Medical Faculty, Inonu University, 34347 Malatya, Turkey; ^4^Department of Pediatric Surgery, Medical Faculty, Inonu University, 34347 Malatya, Turkey

## Abstract

Schwannomas are usually single, encapsulated, and benign tumors of the nerve sheath that arise from the perineural Schwann cells. Schwannomas are mostly seen in the fourth decade. Despite its location in the head and neck region is 25–45%, lip location of schwannoma are very rare. We present a case of a upper lip schwannoma in the pediatric age and review the literature.

## 1. Introduction

Schwannomas or neurilemmomas are especially single, encapsulated, and benign tumors of the nerve sheath that arise from the perineural Schwann cells. Approximately 25–45% of all schwannomas are seen in the head and neck region [1–5]. In the head and neck region, the tumor generally appears in the tongue. Tumor can be less frequently seen in the palate, floor of the mouth, gingiva, and buccal mucosa, parotid gland as well [3, 6, 7]. Upper lip location of the Schwannoma is very rare. It is usually seen in the fourth decade of life [1, 2]. In the literature there is only one reported upper lip schwannoma in the pediatric age group [8]. Schwannoma of the upper lip in a 15-year-old boy is reported with the review of the literature.

## 2. Case Report

**Figure 1:**
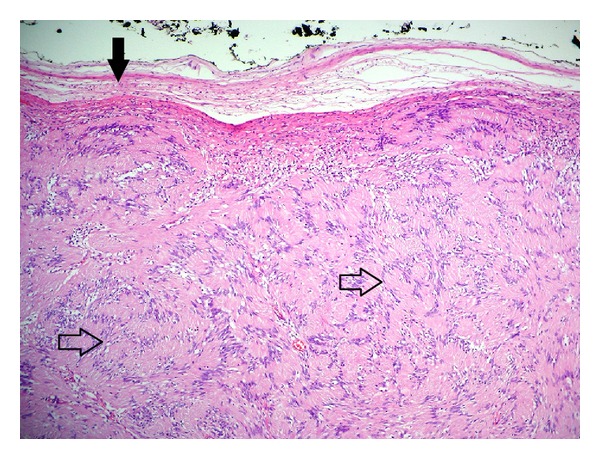
Encapsulated mesenchymal tumor (H&E, ×10): capsule (arrow), Verocay bodies (open arrow).

**Figure 2:**
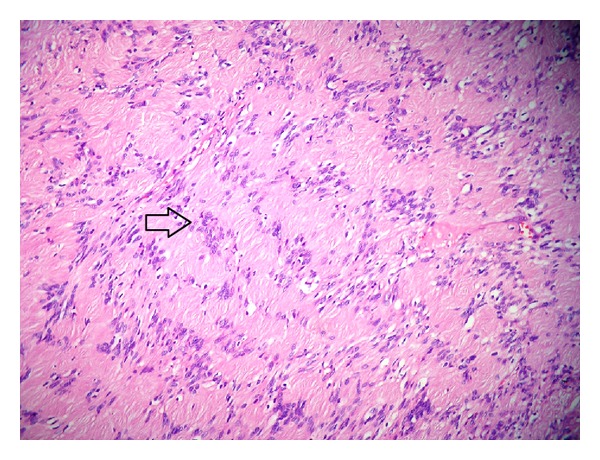
Tumor cells' nuclei arranged in a palisading fashion (Verocay body, open arrow) (H&E, ×20).

A 15-year-old boy referred to Otorhinolaryngology Department of the Inonu University Medical Faculty with a painless mass on the left side of his upper lip. In physical examination, a painless mass of 0,9 × 1,3 cm located in the left portion of upper lip which was mobile, oval shaped, and had smooth surface was revealed. There was neither associated medical findings nor family history about any other concomitant diseases such as neurofibromatosis. He reported no trauma history to the region, as well. The clinical presentation of the mass indicated a benign lesion. The mass was followed up for two years due to the decision of the patient's parents. During the two-year follow-up period, subsequent physical examinations revealed approximately one mm growth of the mass. After two-year follow-up period, the parents consented the mass to be surgically removed. The differential diagnosis included a salivary gland tumor, neurofibroma, or nasolabial cyst. The total excision of the mass was performed under local anesthesia, and the specimen was examined histopathologically. The gross evaluation of the specimen was noted as an encapsulated homogenous mass of 0,9 × 1,3 cm including fibrous connective tissue. The histopathological examination of the excisional biopsy showed an encapsulated cellular mesenchymal tumor that is composed of spindle cells (Figure 1). There was a uniform cellular apperance (Antoni A pattern) through out the tumor without hypocellular areas (Antoni B pattern). The tumor cells were arranged in palisaded fashion (Verocay bodies) (Figure 2). Final pathological diagnosis was made as “schwannoma.” The postoperative course was uneventful, with the patient remaining free of disease after two-year follow-up period.

## 3. Discussion

Schwannomas, also termed as neurilemmomas, are benign nerve sheath tumors that originate from the perineural Schwann cells and can be formed along any sympathetic, cranial, or somatic nerve [9]. They usually arise from peripheral nerves, especially on the deep parts of the soft tissues (the acoustic nerve, posterior spinal root, and the extremities, trunk, body, and neck) [10]. In the head and neck region, the tumor can occur either in peripheral cranial nerves or intracranially. Cranial nerves I and II are not the site of this kind of tumor, because of the absence of Schwann cells [1]. Acoustic nerve is the most common intracranial location. In the head and neck region, the most involved nerves are the vagus and the cervical sympathetic chain [11].

Schwannoma of the head and neck region is predominantly seen in the soft tissues such as tongue, palate, and buccal mucosa [12]. Lip location of this tumor is very rare [2–4]. Schwannoma of the lip was firstly described by Das Gupta et al. in 1969 [13]. Yang and Lin [1] reported their case as upper lip schwannoma and reviewed the literature in 2003. In their report, they documented totally 7 schwannomas of the lip. But, four of those cases had been reported as unknown location [1]. So it was not clear if they have been located on the upper lip or not. The remainder 3 of the 7 cases were on the upper lip region. The literature search revealed 3 new schwannoma of the upper lip between 2003 and 2012 (Table 1). Our case seems to be the second schwannoma of the upper lip in pediatric age group according to the demographic data reported in the literature. Five of 7 cases were female and 2 of them were male (Table 1).

Although schwannomas are typically asymptomatic, sometimes they can be symptomatic according to the nerve origin. The tumor is characterized by a slowly growing solitary mass with a smooth surface, and the structure of the mass can vary from fluctuant cyst to solid [1, 2]. Similarly in our case, the tumor was solid with a slow growth pattern. During the two-year follow-up period, it grew almost only one mm.

Typically schwannoma is an uninodular, encapsulated mass with neural origin histopathologically. The capsule is a thin fibrous tissue, which consists of Antoni type A (cellular palisaded pattern) and Antoni type B (loose poorly cellular pattern) tissues [1, 3, 13]. The histopathological evaluation of our case showed the same features as well.

Asaumi et al. [3] reported characteristic features of schwannomas of the upper lip on ultrasonographic and advanced imaging (computed tomography and magnetic resonance imaging) methods. On ultrasonographic imaging, homogenous and hypoechoic findings and posterior acoustic enhancement were reported. On computed tomography imaging, it has been reported as a definitely marginated mass with homogenous soft-tissue density. Unlike these findings, on magnetic resonance imaging, it has been defined as a homogenous lesion with low-intermediate signal intensity on T1-weighted and high signal intensity on T2-weighted images. But also in this paper authors concluded that radiologic imaging methods should not be considered as routine or necessary for the differential diagnosis, because of the small dimension of this tumor on the upper lip. In this case, we did not perform any radiologic imaging method, because the lesion on the upper lip was relatively small.

The prognosis of schwannoma is quite favorable. If possible, total excision of the lesion with a conservative approach is the treatment of choice. Recurrence is unlikely with complete resection. There is only one case in the literature with recurrence [8]. That case was reported as a multinodular schwannoma with one large and 12 small nodules. Because it was reported as multinodular pattern, that case let us to think about a remaining small nodule in the main area. In our case, complete excision with intraoral vestibular incision was performed. After 1-year follow-up period, our patient had a good clinical course, with no sign of recurrence.

## 4. Conclusion

We presented a rare case of schwannoma in the upper lip in pediatric age group. Schwannoma is a differential diagnosis that should be considered in such cases. The treatment of these tumors is complete resection. These cases usually have good progress, without any recurrence.

## Figures and Tables

**Table 1:** Reported cases of schwannomas of the upper lip.

Authors	Published year	Number of case	Sex	Age	Treatment	Follow-up period	Recurrence
Barbosa and Hansen [4]	1984	1	M	36	Excision	NA	No
Asaumi et al. [3]	2000	1	F	20	NA	NA	No
Yang and Lin [1]	2003	1	F	22	Excision	2 years	No
Yilmaz et al. [12]	2004	1	F	29	Excision	1 year	No
Hashiba et al. [8]	2007	1	F	12	Excision	3 years	Yes
Humber et al. [2]	2011	1	F	82	Excision	5 years	No
Current case		1	M	15	Excision	1 year	No

NA: data not available; M: male; F: female.
